# Crosstalk between Chemokine Receptor CXCR4 and Cannabinoid Receptor CB_2_ in Modulating Breast Cancer Growth and Invasion

**DOI:** 10.1371/journal.pone.0023901

**Published:** 2011-09-07

**Authors:** Mohd W. Nasser, Zahida Qamri, Yadwinder S. Deol, Diane Smith, Konstantin Shilo, Xianghong Zou, Ramesh K. Ganju

**Affiliations:** Department of Pathology and Comprehensive Cancer Center, The Ohio State University, Columbus, Ohio, United States of America; University of Nebraska Medical Center, United States of America

## Abstract

**Background:**

Cannabinoids bind to cannabinoid receptors CB_1_ and CB_2_ and have been reported to possess anti-tumorigenic activity in various cancers. However, the mechanisms through which cannabinoids modulate tumor growth are not well known. In this study, we report that a synthetic non-psychoactive cannabinoid that specifically binds to cannabinoid receptor CB_2_ may modulate breast tumor growth and metastasis by inhibiting signaling of the chemokine receptor CXCR4 and its ligand CXCL12. This signaling pathway has been shown to play an important role in regulating breast cancer progression and metastasis.

**Methodology/Principal Findings:**

We observed high expression of both CB_2_ and CXCR4 receptors in breast cancer patient tissues by immunohistochemical analysis. We further found that CB_2_-specific agonist JWH-015 inhibits the CXCL12-induced chemotaxis and wound healing of MCF7 overexpressing CXCR4 (MCF7/CXCR4), highly metastatic clone of MDA-MB-231 (SCP2) and NT 2.5 cells (derived from MMTV-neu) by using chemotactic and wound healing assays. Elucidation of the molecular mechanisms using various biochemical techniques and confocal microscopy revealed that JWH-015 treatment inhibited CXCL12-induced P44/P42 ERK activation, cytoskeletal focal adhesion and stress fiber formation, which play a critical role in breast cancer invasion and metastasis. In addition, we have shown that JWH-015 significantly inhibits orthotopic tumor growth in syngenic mice *in vivo* using NT 2.5 cells. Furthermore, our studies have revealed that JWH-015 significantly inhibits phosphorylation of CXCR4 and its downstream signaling *in vivo* in orthotopic and spontaneous breast cancer MMTV-PyMT mouse model systems.

**Conclusions/Significance:**

This study provides novel insights into the crosstalk between CB_2_ and CXCR4/CXCL12-signaling pathways in the modulation of breast tumor growth and metastasis. Furthermore, these studies indicate that CB_2_ receptors could be used for developing innovative therapeutic strategies against breast cancer.

## Introduction

Cannabinoids exert their effects by binding with two heptahelical Gα_i/_Gα_o_-protein-coupled receptors, CB_1_ and CB_2_. CB_1_ receptors are expressed predominantly in the central nervous system, whereas CB_2_ receptor is mainly expressed by the cells of the immune system [1], [2]. Many selective agonists which have significantly higher affinities for their specific receptors have been developed. CB_1_-specific agonists include synthetic cannabinoids methanandamide (Met-f-AEA), arachidonylcyclopropylamide (ACPA), and arachidonyl-2-chloroethylamide (ACEA), whereas synthetic cannabinoids JWH-015 and JWH-133 specifically bind to cannabinoid receptor CB_2_. Cannabinoids have been shown to inhibit proliferation and growth of various cancers including breast, liver, prostate, skin, and lung in *in vitro* and *in vivo* mouse models [3], [4], [5], [6], [7]. Cannabinoids have also been shown to directly induce apoptosis by causing cell cycle arrest in neoplastic cells [8], [9]. Furthermore, experimental evidence has shown that cannabinoids may also inhibit angiogenesis *in vitro* and *in vivo* [3], [5], [7], [10], [11], [12], [13]. Although cannabinoid receptors have been shown to modulate several signaling pathways involved in the control of cell survival, not much is known about their role in the regulation of chemokine receptor CXCR4-mediated signaling in tumors [5], [6], [14].

Chemokine receptor CXCR4 is a seven membrane spanning G-protein coupled receptor and has been shown to be overexpressed on various malignant cancers including breast cancer [15], [16]. CXCR4 binds to its cognate ligand CXCL12, also known as stromal-derived factor 1-α (SDF1α). The CXCR4/CXCL12 receptor axis has been shown to play an important role in metastasis of CXCR4-expressing tumor cells to organs and tissues that produce high amounts of CXCL12 such as the liver, bone, lymph nodes, lungs, and brain [17], [18], [19]. Overexpression of CXCR4 is associated with metastasis and poor prognosis of breast cancer [20], [21], [22], [23], [24], [25]. CXCR4/CXCL12 axis has been shown to enhance tumor growth by modulating tumor stroma through activation of cancer-associated fibroblasts and recruitment of CXCR4-positive endothelial precursor cells, thereby enhancing angiogenesis [26]. Targeting CXCR4 with neutralizing antibodies and small molecule antagonists has been shown to inhibit breast tumor growth and metastasis *in vitro* and *in vivo* [19], [27], [28], [29], [30].

CXCL12/CXCR4 signaling axis has also been shown to modulate focal adhesion complexes which regulate cell migration [31]. Focal adhesions are the primary links between the cell and the extracellular matrix (ECM), formed by focal adhesion kinase (FAK) and vinculin, which connect integrins to the actin cytoskeleton [32]. Appropriate regulation of fiber association and disassociation, mediated by FAK and vinculin, is important for controlling cellular migration and signaling [32]. Actin stress fibers are anchored in focal adhesions and are responsible for cell traction and ECM reorganization [33].

In the present study, we have analyzed the effects of synthetic non-psychoactive cannabinoid JWH-015 that specifically binds to CB_2_ receptors on breast cancer and have shown that this compound inhibits CXCL12/CXCR4-induced breast cancer invasive properties *in vitro*. In addition, we have shown that it inhibits growth in *in vivo* mouse model systems. Furthermore, our signaling studies in breast cancer cell lines as well as in tumors derived from experimental mice have revealed that synthetic cannabinoids inhibit tumorigenesis by suppressing the phosphorylation of CXCR4 and its downstream target, ERK. This study provides novel insights about anti-tumorigenic effects of CB_2_ receptors in breast cancer through modulation of CXCR4/CXCL12 signaling axis. Furthermore, these studies suggest that CB2 could be developed as a potential therapeutic target against breast cancer growth and metastasis.

## Materials and Methods

### Reagents and antibodies

Cell culture reagents were purchased from Gibco Laboratories (Grand Island, NY). The following reagents and antibodies used in this study were purchased from different sources: anti-CB_2_ (Affinity Bioreagents, Golden, CO, USA); JWH-015 (Tocris Cookson, Ellisville, MO); human CXCL12 and murine CXCL12 (Peprotech); vinculin (Sigma); pCXCR4/CXCR4 (Abcam); pERK/ERK (Santa Cruz); Phalloidin-568 (Invitrogen); and Ki67 (NeoMarkers).

### Cell culture

MCF-7/CXCR4 (kindly provided by Dr. Ann Richmond, Vanderbilt University School of Medicine, Nashville, TN) [34] and SCP2, a subclone of MDA-MB-231 cells (kindly provided by Dr. Joan Massagué, Memorial Sloan-Kettering Cancer Center, New York, NY) [35], and NT2.5 cells (obtained from Dr. Gustavo Leone laboratory, The Ohio State University) [36] were cultured in DMEM containing 10% heat-inactivated fetal bovine serum (FBS), 5 units/mL penicillin, and 5 mg/mL streptomycin.

### Western blot analysis

Cells plated in 100 cm^2^ dishes were lysed in lysis buffer (50 mM Tris–HCl, 150 mM NaCl, 0.5% Triton X-100, 0.5% deossicolic acid, 10 mg/ml leupeptin, 2 mM phenylmethylsulfonyl fluoride, 10 mg/ml aprotinin) and tumor samples were homogenized in cell lysis buffer (Cell Signaling Technologies, Beverly, MA, USA). 50 µg of proteins was loaded on 4–12% SDS–polyacrylamide gels (Invitrogen) under reducing conditions, transferred to nitrocellulose membranes (BioRad) and blocked with 5% milk. Membranes were incubated overnight with primary antibody, washed three times, and incubated for 1 h at RT with horseradish peroxidase-conjugated secondary antibody. The membranes then were washed and stained using a chemiluminescence system (ECL-Amersham Biosciences) and exposed to X-ray film (Kodak).

### Confocal Microscopy

Confocal microscopy was carried out as described previously [4], [5]. Briefly, cells were plated on 2-mm glass coverslips coated with 15 mg/ml of poly-L-lysine and cultured in complete DMEM for 24 h. After treatment with JWH-015 and stimulation with CXCL12, cells were fixed with 4% paraformaldehyde, blocked with 5% normal goat serum for 60 min, incubated in FITC-labeled anti-vinculin (Sigma) or Phalloidin-568 for 2 h at RT. Images were acquired using Leica Axiovert S100 TV microscope (Carl Zeiss, Oberkochen, Germany).

### Chemotactic Assays

Chemotactic assays were performed using transwell chambers (Costar 8.0 µm pore size) as described previously [4], [5]. Briefly, serum starved MCF-7/CXCR4, SCP2 and NT2.5 cells were pretreated with JWH-015 or vehicle (20 µM) overnight. Top chambers were loaded with 150 µL of 1×10^6^ cells/ml in serum-free medium and bottom chambers contained serum-free medium in the presence or absence of CXCL12 (100 ng/ml). Cells that migrated across the membrane after 6 h were counted by fixing in 37% formaldehyde and 25% glutaraldehyde in PBS and staining with 0.1% crystal violet in PBS for 30 minutes. The number of migratory cells per membrane was measured by light microscopy, counted in 5 fields and the percentage of migration determined.

### Wound healing Assay

Wound healing assays were performed as described previously [4]. Briefly, cells were grown to 70% confluence in complete DMEM. Monolayers were wounded by scratching with a sterile plastic 200 µL micropipette tip, washed, and incubated in DMEM with 0.1% FBS in the presence or absence of JWH-015 or vehicle and CXCL12 (100 ng/ml). After 18 or 24 h, cells were fixed with 4% paraformaldehyde in PBS for 5 min at RT and photographed using a low-magnification phase-contrast microscope. The extent of migration into the wound area was evaluated qualitatively using ImageJ software.

### Flow cytometry assay

Cells were grown in 6-well plates for 24 h, incubated in 2 mM EDTA, removed from plates, and fixed with ice cold 70% ethanol in PBS. After centrifugation, pellets were resuspended and stained with primary antibodies for 30 min. Samples were analyzed by flow cytometry (BD FACS Calibur) at RT in the dark.

### Animal Studies

*In vivo* experiments were done in compliance with the guidelines and protocols approved by Institutional Animal Care and Use Committee of The Ohio State University (2007A0233-R1). FVB mice (Charles River Laboratories Inc.) were used for orthotopic tumor studies at 4 to 6 wk of age. Tumors were induced by injecting NT 2.5 cells 3×10^6^ (100 µL) into the #4 mammary gland of FVB mice. When tumors were palpable, the animals were assigned randomly to various groups (n = 5) and injected peritumorally with JWH-015 (5 mg/kg) or vehicle on alternate days for 4 wk. Tumors were measured every wk with external calipers and tumor volume was calculated according to the formula *V* = 0.52×*a* ^2^×*b*, where *a* is the smallest superficial diameter and *b* is the largest superficial diameter [5], [37].

Female heterozygous PyMT mice (n = 5) were treated either with JWH-015 (5 mg/kg body weight) or vehicle, daily for 28 days. After 8 wk, mice were sacrificed and tumors were dissected, weighed, and divided into pieces, which were snap frozen in liquid nitrogen for protein analysis [5].

### Immunohistochemistry

Tissue microarrays from paraffin-embedded formalin fixed breast cancer tissue specimens were prepared as described earlier [5]. These tissue microarrays were obtained from the Human Tissue Resource Network, Department of Pathology, The Ohio State University. Samples were stained with CXCR4 antibody (Abcam) in a 1∶100 dilution and CB_2_ antibody (Affinity Bio-Reagent) in a 1∶200 dilution and detected using a kit (Vector Laboratories) as described earlier [5]. Slides were visualized with 3,3′-diaminobenzidine chromogen for the intensity and analysis of CXCR4 and CB_2_ expression.

Samples from tumor xenografts of mice were dissected, embedded in OCT (Tissue-Tek) and stained using standard immunohistochemistry techniques as per the manufacturer's recommendation (Vector Laboratories), using the primary antibody against Ki-67 (Neomarkers) at a dilution of 1∶100. Slides were stained with secondary antibodies and detected as described earlier [4], [5].

### Statistical analysis

Student's two-tailed *t* test was used to compare vehicle and cannabinoid-treated groups. p<0.05 was considered to be statistically significant. For all graphs, ^*^indicates *P*<0.05; ^**^indicates *P*<0.01.

## Results

### Primary breast cancer tissues express CB_2_ and CXCR4

**Figure 1:**
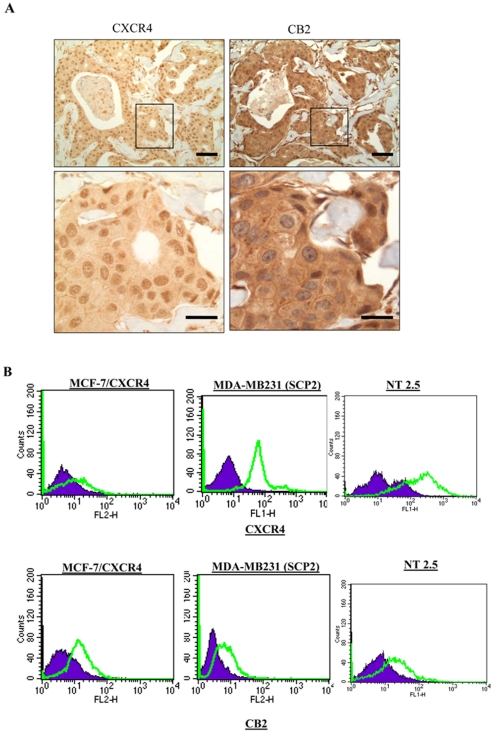
Expression of CXCR4 and CB_2_ receptors in primary breast cancer tissues and cell lines. (A) Representative photomicrographs of immunohistochemical staining of primary breast cancer tissues showing CXCR4 and CB_2_ staining. Scale bars 50 µm and 200 µm. (B). FACS analysis of cell surface expression of CXCR4 and CB_2_ in MCF-7/CXCR4, SCP2 and MMTV-*neu* (NT 2.5) cells.

Cannabinoids have been shown to exert their effects through cannabinoid receptors CB_1_ and CB_2_. However, not much is known about the role of CB_2_ receptors in modulating CXCR4-mediated effects in breast cancer. To elucidate the role of CB_2_ and CXCR4 in breast cancer, we assessed their expression in tumor samples from 82 breast cancer patients using tissue microarrays. We observed expression of CB_2_ (58%) and CXCR4 (90%) in these cases (Fig. 1A). In addition, MCF-7/CXCR4 (MCF-7 overexpressing CXCR4), SCP2 (highly metastatic MDA-MB-231 clone), and NT 2.5 cells (derived from MMTV-neu mice) were shown to express both CXCR4 and CB_2_ (Fig. 1B) by Flow cytometry.

### CB_2_ agonist JWH-015 inhibits CXCL12-induced migration and invasion of breast cancer cells

**Figure 2:**
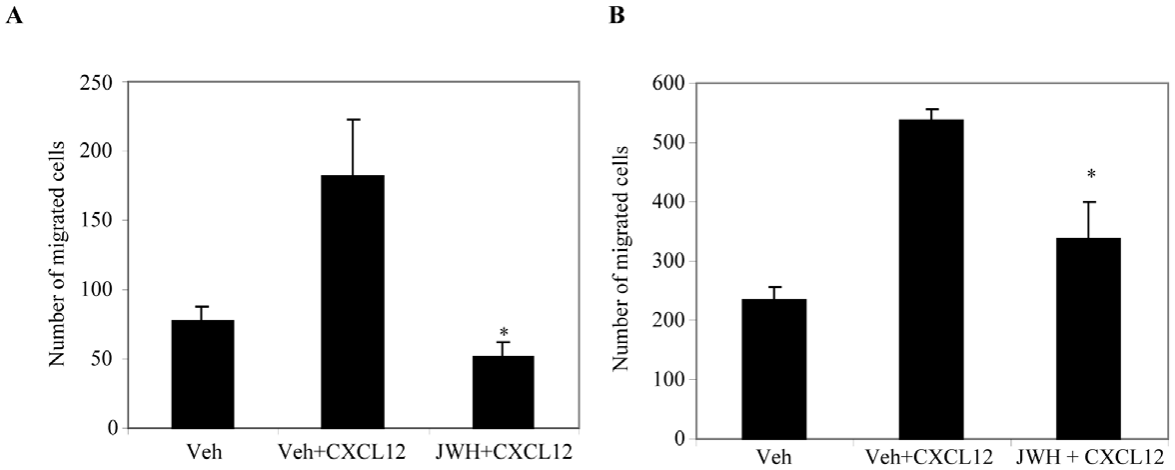
CB_2_ agonist inhibits CXCL12-induced cell migration in breast cancer cells. Breast cancer cells (A) MCF-7/CXCR4-WT and (B) MDA-MB231 (SCP2) were pretreated overnight with vehicle or JWH-015 (20 µM) and 1×10^5^ cells were plated on the top chamber of 8 µm pore polycarbonate membrane filters and medium in absence or presence of CXCL12 (100 ng/ml) was placed in the lower chamber. After 12 hours of incubation, cells that migrated across the filter towards medium with or without CXCL12 (100 ng/ml) were fixed, stained and counted by bright-field microscopy in five random fields. Data represent the mean ± SD, representative experiments (n = 3) are shown. *P<0.05 vs. vehicle. Veh: Vehicle.

**Figure 3:**
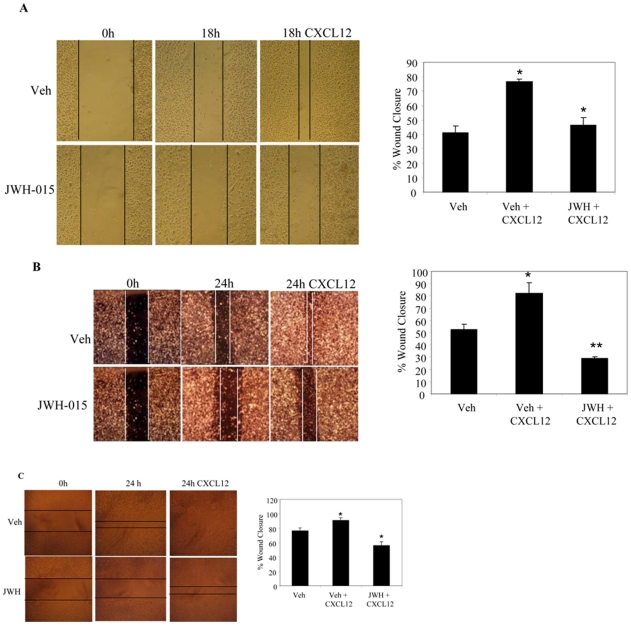
CB_2_ agonist inhibits CXCL12-induced wound healing in breast cancer cells. Breast cancer cells (A) MCF-7/CXCR4-WT, (B) MDA-MB231 (SCP2), and (C) MMTV-*neu* (NT 2.5, murine mammary cells) were grown for confluence in complete medium in six-well plates and then scratched with a 200 µl pipette tip to make wounds. The cells were treated with vehicle or JWH-015 (20 µm) and the closure of the wounds was monitored in presence or absence of CXCL12 (100 ng/ml) by microscopy after 18 or 24 hrs. Quantitative analysis of % wound closure as shown in left panels was determined my ImageJ software. Data represent the mean ± SD, representative experiments (n = 3) are shown. *P<0.05 vs. vehicle; **P<0.005 vs. vehicle. Veh: Vehicle.

CXCR4/CXCL12 signaling axis has been shown to promote migration and invasion of breast cancer cells to distant sites of metastasis such as the lung, brain, bone, lymph nodes, and liver [19], [31], [38]. Hence, to evaluate the potential of CB_2_ as a possible therapeutic target, the effect of CB_2_ agonist JWH-015 on CXCL12-induced cell migration was investigated. As shown in Fig. 2, JWH-015 (20 µM) showed significant inhibition of CXCL12-induced migration of MCF-7/CXCR4 and SCP2 cells. We further analyzed the effect of JWH-015 on CXCL12-induced invasion using wound healing assays. We observed a significant inhibition of CXCL12-induced wound healing in MCF-7/CXCR4, SCP2, and NT 2.5 after overnight pretreatment with JWH-015 (20 µM) compared to the vehicle-treated cells (Fig. 3). Thus these results suggest that CB_2_ receptor specific agonists significantly reduced CXCL12/CXCR4-induced migration and invasion of breast cancer cells.

### CB_2_ agonist JWH-015 inhibits CXCR4/CXCL12-mediated signaling in breast cancer cells

**Figure 4:**
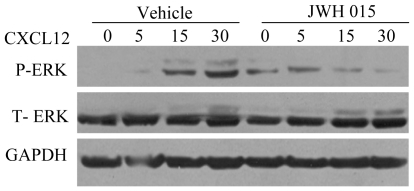
CB_2_ agonist inhibits CXCL12-induced ERK phosphorylation. MDA-MB231 (SCP2) cells were treated overnight with vehicle or JWH-015 (20 µm) and stimulated with CXCL12 (100 ng/ml) for different time periods. Cell lysates were analyzed for Phospho-ERK (p-ERK), ERK and GAPDH by Immunoblotting.

CXCR4/CXCL12 has been shown to induce growth and migration of different types of cells by activating various signaling pathways including activation of p44/p42 ERK [15], [34], [39]. We analyzed the effect of JWH-015 on CXCL12-induced signaling and observed that it inhibits phosphorylation of p44/p52 MAPK in SCP2 cells (Fig. 4).

**Figure 5:**
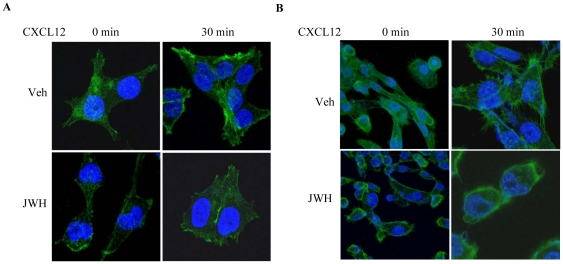
CB_2_ agonist inhibits CXCL12-induced focal adhesions and stress fibres formation. Confocal microscopic visualization of (A) MCF-7/CXCR4 for focal adhesion (stained for Vinculin) or (B) SCP2 cells for stress fibers (stained for phalloidin). The cells were pretreated with vehicle or JWH-015 (20 µm) overnight and then stimulated with CXCL12 (100 ng/ml; 30 min).

CXCL12 has also been shown to modulate focal adhesion complexes which regulate cell migration [31]. We analyzed the effect of JWH-015 on CXCL12-induced focal adhesion formation and actin stress fiber formation in various breast cancer cell lines. As shown in Fig. 5A, JWH-015 inhibited focal adhesion formation as detected by changes in vinculin on the cellular membrane using immunofluorescence in MCF-7/CXCR4 cells. In addition, we observed that JWH-015 inhibited CXCL12-mediated actin stress fiber formation in SCP2 cells (Fig. 5B). These studies indicate that CB_2_-specific agonists may modulate cell migration by inhibiting actin stress fiber and focal adhesion formation.

### CB_2_ agonist JWH-015 inhibits tumor growth in syngenic mouse models by downregulation of CXCR4-mediated signaling

**Figure 6:**
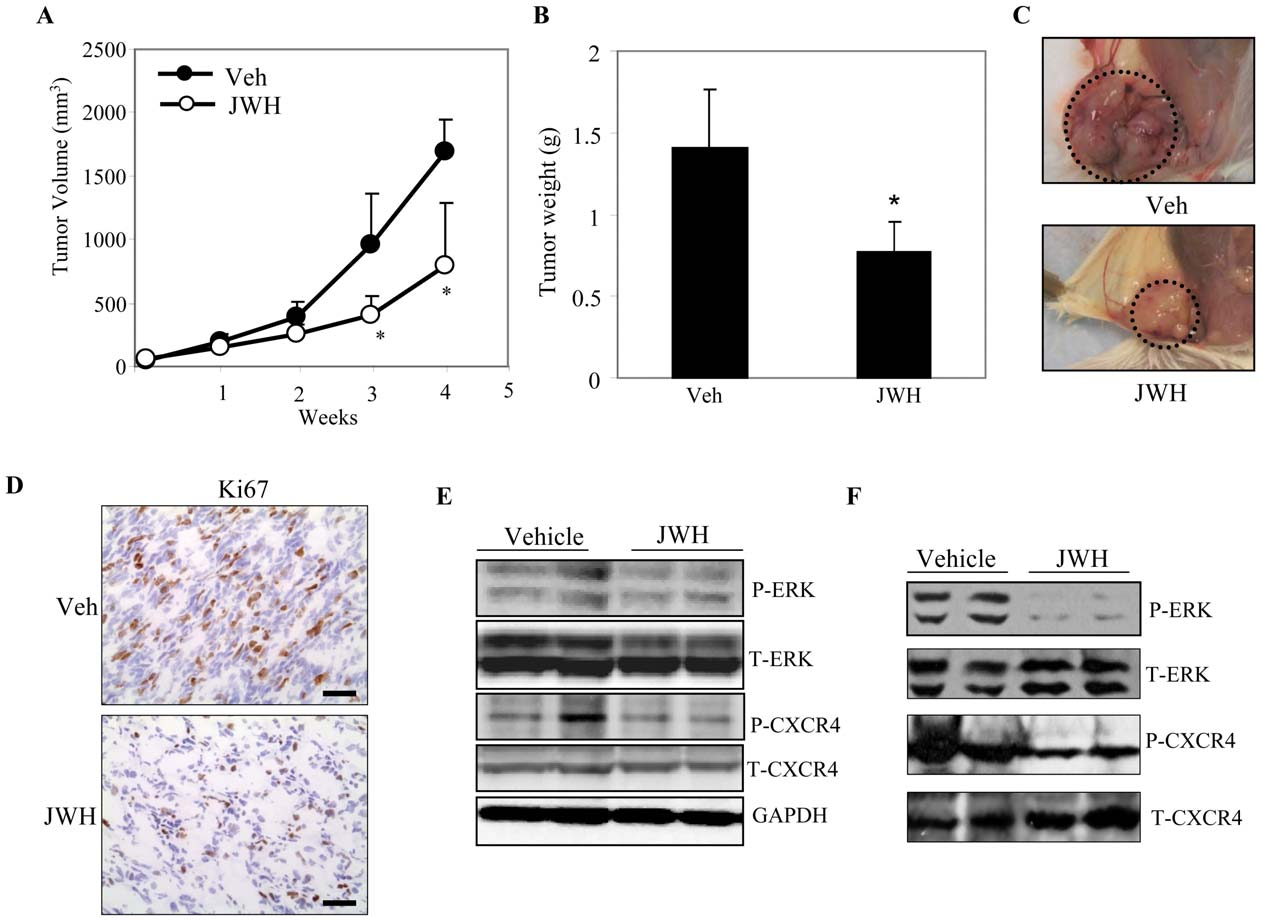
CB_2_ agonist inhibits tumor growth in syngenic mouse models. NT 2.5 cells (2×10^6^ in 100 ul PBS) were implanted orthotopically into mammary gland (#4) of mice. Experimental mice (n = 5) were treated peritumorally either with JWH-015 (5 mg/kg body wt) or vehicle on alternate days for 28 days starting 14 days after injection of the cells. (A) Tumors were measured every wk with external calipers and tumor volume was calculated according to the formula *V* = 0.52×*a* ^2^×*b*, where *a* is the smallest superficial diameter and *b* is the largest superficial diameter. (B) After 28 days, the tumors were excised and weighed. (C) A representative photograph of mice showing tumors dissected from vehicle or JWH-015 treated groups. (D) Representative photomicrographs of immunostaining with Ki67 (proliferation marker) of tumors extracted from JWH-015 treated mice compared to vehicle treated. Scale bars, 50 µm. (E) Tumor lysates from mice treated with JWH or vehicle were analyzed for Phospho-CXCR4 (pCXCR4), CXCR4, Phospho-ERK (pERK), ERK and GAPDH by Immunoblotting. (F) Tumor lysates derived from PyMT transgenic mice treated with JWH or vehicle and analyzed for pERK, ERK, pCXCR4 and CXCR4 by Immunoblotting. *P<0.05 vs. vehicle. Veh: Vehicle.

To further analyze the effect of CB_2_ agonists on *in vivo* growth and proliferation, we induced tumors by injecting NT 2.5 cells into syngenic (FVB) mice and treated them with JWH-015 or vehicle for 4 wk. NT 2.5 cells have been derived from MMTV-*neu* mouse models (FVB) and have been used for analyzing breast tumor growth in syngenic mouse models [36]. We observed a significant inhibition of tumor formation in JWH-015 treated mice compared to the vehicle treated group (Fig. 6A to C). In addition, there was a significant decrease in proliferation as indicated by downregulation of Ki67 (proliferation marker) immunostaining in JWH-015-treated compared to the vehicle-treated tumors (Fig. 6D) [40]. To further elucidate the mechanism of inhibition, we analyzed the tumors extracted from JWH-015 or vehicle treated mice. We observed a significant decrease in the phosphorylation of CXCR4 and ERK in tumors treated with JWH-015 compared to vehicle treated tumors (Fig. 6E). However, no change in total protein levels of ERK and CXCR4 were observed in JWH-015 compared to vehicle-treated tumors.

We have previously reported a delay and inhibition of highly aggressive spontaneous mammary tumor formation in transgenic PyMT mice after treatment with JWH-015 compared to the vehicle-treated group [5]. To further confirm the effect of JWH-015 on phosphorylation of ERK and CXCR4 *in vivo*, we analyzed the cell lysates of tumors derived from JWH-015 or vehicle-treated PyMT mice. We observed a significant decrease in the phosphorylation of CXCR4 and ERK in tumors extracted from JWH-015 treated mice compared to the vehicle treated group (Fig. 6F). Taken together, these results suggest that JWH-015 inhibits tumor growth *in vivo*. Furthermore, these studies indicate that the inhibition of tumorigenesis may be caused by a decrease of CXCR4 phosphorylation that may lead to lower ERK phosphorylation and thereby reduce proliferation.

## Discussion

Breast cancer is the second leading cause of cancer death in women in the United States. Despite recent advances in hormonal therapies, mortality still remains high due to breast cancer metastasis to other organs. Synthetic cannabinoids that bind to cannabinoid receptors CB_1_ and CB_2_ have been shown to inhibit migration, metastasis, and invasion of various cell types including breast cancer cells [4], [5], [6], [37], [41]. However, not much is known about the mechanisms by which CB_1_ and CB_2_ mediate their inhibitory effects. The majority of breast cancers have been shown to overexpress chemokine receptor CXCR4, which has been correlated with poor prognosis [34]. Furthermore, high CXCR4 expression was also correlated to poor clinical outcome in triple negative breast cancers, which are difficult to treat [39], [42]. Here, we report for the first time that CB_2_ specific synthetic agonist inhibits CXCL12-induced migration and invasion of breast cancer cell lines *in vitro*. Furthermore, we have shown that this compound inhibits tumor growth *in vivo* and downmodulates CXCR4 phosphorylation and downstream signaling.

We observed expression of CXCR4 (90%) and CB_2_ (58%) in primary human tumor breast cancer samples by tissue microarray analysis. We also found that both CXCR4 and CB_2_ receptors are expressed on various breast cancer cell lines, including the highly invasive triple negative breast cancer cell line SCP2. Although CXCR4 and CB_2_ have previously been shown to be expressed by breast cancer tissues, this is the first report showing co-expression of these receptors on breast cancer tissues and cells.

CXCR4 and its cognate ligand CXCL12 have been shown to play an important role in regulating metastasis of breast cancer to specific organs [19], [39], [43]. Our results show that CB_2_ agonist JWH-015 significantly inhibits CXCL12-induced cell migration and wound healing in breast cancer cells. Previously, CB_2_ agonists have been shown to modulate CXCL12-induced migration of T-cells [44]. Wound healing and cell migration have been shown to play an important role in regulating breast cancer cell metastasis. These results suggest that CB_2_ receptor activation may modulate breast cancer metastasis by inhibiting the CXCR4/CXCL12 signaling axis.

The molecular mechanisms involved in CB_2_-mediated inhibition of migration/invasion induced by CXCR4/CXCL12 are not well characterized. We have shown that JWH-015 inhibits CXCL12-induced phosphorylation of p44/p42 ERK in breast cancer cells. CXCL12 has been shown to induce various signaling pathways including p44/p42 ERK in breast cancer and other cell types [15], [34], [39], [45], [46], [47], [48], [49]. Previously, ERK has been shown to regulate migration of several cell types [47], [50]. We also observed that JWH-015 inhibits CXCL12-induced focal adhesion formation along with a decrease in actin stress fibers in breast cancer cells. Focal adhesions and actin stress fibers have been shown to play an important role in cell migration and metastasis [51], [52]. Focal adhesions contain transmembrane integrin receptors that join the ECM to actin stress fibers [32], [33], [51]. Actin stress fibers are anchored in focal adhesions and are responsible for cell traction and ECM reorganization [33]. The actin cytoskeleton plays an important role in defining cell shape, morphology, and regulating cellular migration. FAK and vinculin are responsible for focal adhesion turnover and can be monitored for altered cellular behavior [32], [51]. Inhibition of FAK and vinculin causes a significant decrease in normal cell spreading and migration of breast cancer cells [32]. Increased actin stress fiber polymerization has been correlated with enhanced cell wound healing and motility [53]. Our results suggest that JWH-015-mediated effects of p44/p42 ERK, focal adhesion formation, and actin stress fiber polymerization may lead to reduced CXCL12-induced motility and wound healing capability of breast cancer cells.

Mouse models are valuable tools for exploring and understanding molecular mechanisms of breast cancer progression and metastasis. In this study we used syngenic MMTV-neu mouse models to analyze the effect of JWH-015 on breast cancer tumor growth *in vivo*. We observed a significant reduction in orthotopic tumor growth in mice treated with JWH-015 compared to vehicle controls. These studies are consistent with previous studies using PyMT and MMTV-neu transgenic mouse models for breast cancer [5], [41]. We showed that JWH-015 significantly inhibits breast cancer growth and proliferation in mice injected with NT 2.5 cells. Further, analyses of tumors derived from syngenic and PyMT transgenic mouse models indicated that JWH-015 inhibited phosphorylation of CXCR4 and ERK without decreasing total protein expression. This is the first report indicating that CB_2_ agonist JWH-015 inhibits CXCR4 phosphorylation *in vivo*. These studies suggest that CB_2_ may crosstalk with CXCR4 receptors to downregulate their signaling mechanisms.

The results of this study suggest that CB_2_-specific non-psychoactive synthetic cannabinoid JWH-015 inhibits CXCL12-induced migration and invasive properties of breast cancer cells. Furthermore, elucidation of signaling mechanisms reveals that JWH-015 inhibits CXCL12-induced CXCR4 and ERK phosphorylation, focal adhesion formation and actin stress fiber polymerization. Thus, we conclude that CB_2_-specific synthetic cannabinoids that do not possess psychoactivity can be developed to design novel therapies against breast cancer growth and metastasis by blocking CXCR4/CXCL12-induced signaling.
